# Staphylococcus aureus Infects Osteoclasts and Replicates Intracellularly

**DOI:** 10.1128/mBio.02447-19

**Published:** 2019-10-15

**Authors:** Jennifer L. Krauss, Philip M. Roper, Anna Ballard, Chien-Cheng Shih, James A. J. Fitzpatrick, James E. Cassat, Pei Ying Ng, Nathan J. Pavlos, Deborah J. Veis

**Affiliations:** a Division of Bone & Mineral Diseases, Musculoskeletal Research Center, Washington University School of Medicine, Saint Louis, Missouri, USA; b Washington University Center for Cellular Imaging, Washington University School of Medicine, Saint Louis, Missouri, USA; c Department of Pathology, Microbiology, and Immunology, Vanderbilt University Medical Center, Nashville, Tennessee, USA; d Department of Pediatrics, Division of Pediatric Infectious Diseases, Vanderbilt University Medical Center, Nashville, Tennessee, USA; e Department of Biomedical Engineering, Vanderbilt University Medical Center, Nashville, Tennessee, USA; f Vanderbilt Institute for Infection, Immunology and Inflammation (VI4), Vanderbilt University Medical Center, Nashville, Tennessee, USA; g Vanderbilt Center for Bone Biology, Vanderbilt University Medical Center, Nashville, Tennessee, USA; h School of Biomedical Sciences, University of Western Australia, Perth, Western Australia, Australia; i Department of Pathology and Immunology, Washington University School of Medicine, Saint Louis, Missouri, USA; j Shriners Hospitals for Children, Saint Louis, Missouri, USA; Georgia Institute of Technology School of Biological Sciences

**Keywords:** *Staphylococcus aureus*, bone, osteoclasts, osteomyelitis, RANKL, intracellular bacteria

## Abstract

Osteomyelitis (OM), or inflammation of bone tissue, occurs most frequently as a result of bacterial infection and severely perturbs bone structure. OM is predominantly caused by Staphylococcus aureus, and even with proper treatment, OM has a high rate of recurrence and chronicity. While S. aureus has been shown to infect osteoblasts, it remains unclear whether osteoclasts (OCs) are also a target of intracellular infection. Here, we demonstrate the ability of S. aureus to intracellularly infect and divide within OCs. OCs were differentiated from bone marrow macrophages (BMMs) by exposure to receptor activator of nuclear factor kappa-B ligand (RANKL). By utilizing an intracellular survival assay and flow cytometry, we found that at 18 h postinfection the intracellular burden of S. aureus increased dramatically in cells with at least 2 days of RANKL exposure, while the bacterial burden decreased in BMMs. To further explore the signals downstream of RANKL, we manipulated factors controlling OC differentiation, NFATc1 and alternative NF-κB, and found that intracellular bacterial growth correlates with NFATc1 levels in RANKL-treated cells. Confocal and time-lapse microscopy in mature OCs showed a range of intracellular infection that correlated inversely with S. aureus-phagolysosome colocalization. The propensity of OCs to become infected, paired with their diminished bactericidal capacity compared to BMMs, could promote OM progression by allowing S. aureus to evade initial immune regulation and proliferate at the periphery of lesions where OCs are most abundant.

## Abstract (precis)

The inflammation of bone tissue is called osteomyelitis, and most cases are caused by an infection with the bacterium Staphylococcus aureus. To date, the bone-building cells, osteoblasts, have been implicated in the progression of these infections, but not much is known about how the bone-resorbing cells, osteoclasts, participate. In this study, we show that S. aureus can infect osteoclasts and proliferate inside these cells, whereas bone-residing macrophages, immune cells related to osteoclasts, destroy the bacteria. These findings elucidate a unique role for osteoclasts to harbor bacteria during infection, providing a possible mechanism by which bacteria could evade destruction by the immune system.

## INTRODUCTION

Although osteomyelitis (OM) technically refers to the inflammation of the marrow cavity, it is most frequently used to indicate infection of the bone itself. At the center of infectious OM lesions, bone is frequently lost by necrosis, forming a devascularized segment of bone known as a sequestrum. Osteoclasts (OCs) are recruited to the site via inflammatory cytokine release and expand the area of bone loss. New bone is formed on the periosteum in the body’s attempt to isolate the infection, generating an involucrum. Thus, the normal balance between bone formation and resorption is disrupted in OM, leading to pathological fractures and deformities (1). Compared to other tissues, bone infections are especially damaging and intractable, with treatment often involving prolonged antibiotics paired with surgical debridement (2). However, even with proper treatment, OM has a high recurrence rate, leading to a chronic debilitating condition (3, 4). OM is predominantly divided into two broad categories: (i) acute hematogenous OM caused by bacteria seeding directly into bone from the circulation, and (ii) secondary OM originating from a contiguous source like a soft tissue infection or orthopedic implant or following open fracture (1, 5). Acute hematogenous OM is most common in children, with 85% of cases occurring in children under 17 years of age, whereas secondary OM infections are more common in older adults, especially those undergoing orthopedic surgery (5, 6), and they account for a projected cost of more than $1 billion dollars annually in the United States (7). Regardless of the type of OM, most cases are caused by Staphylococcus aureus (6, 8).

Despite the clinical significance of OM infections, there remains a dearth of knowledge as to the mechanisms underlying the etiopathology of the disease. Patient-centered studies have demonstrated the paramount role of biofilms in seeding implants and driving the subsequent chronicity and intractability of the infections (7, 9). Similar studies have examined the localization of bacteria in the bone tissue, including within osteoblasts, osteocytes, and bone canaliculi, and characteristics of the inflammatory response initiated by infection (10–13). At this point, most of the basic science work on OM has focused on characterizing changes to the structure of the bone (10, 14–16), elucidating bacterial survival strategies (17, 18), or examining the role of osteoblasts in promoting infection (17, 19–21). *In vitro*, S. aureus has the ability to infect osteoblasts, persist intracellularly, and induce the release of osteoclastogenic and inflammatory cytokines (15, 19, 20, 22). While it is not clear how many osteoblasts in an OM lesion harbor intracellular bacteria *in vivo*, cytokines are produced locally (23–28). Investigation into the role of OCs in OM has focused mainly on determining the effects of S. aureus infection on bone resorption, often overlooking whether OCs could also be a direct target of infection (29–32).

OCs are differentiated from the myeloid lineage in a process that involves receptor activator of nuclear factor kappa-B ligand (RANKL) signaling to alternative NF-κB signaling through NF-κB-inducing kinase (NIK) and RelB, activating NFATc1 (33–35). However, despite their shared lineage, OCs show a decreased release of inflammatory cytokines and nitric oxide when challenged with bacteria compared to macrophages (31). Most macrophages attempt to destroy internalized S. aureus via acidification of the phagolysosome, digestive hybrid organelles formed upon fusion of phagosomes with phagolysosomes (36). However, S. aureus has been shown to persist within some peripheral macrophages through intraphagolysosomal replication and escape (37, 38). Yet, the ability of OCs to control S. aureus infection is not known. In this study, we show that S. aureus is not only able to infect OCs but also proliferates within them, avoiding lysosomal compartments. This study illuminates a novel function of OCs in OM as an intracellular reservoir allowing bacterial proliferation, in addition to their ability to modulate bone structure through perturbed remodeling.

## RESULTS

### S. aureus resides within osteoclasts *in vivo* and *in vitro*.

**FIG 1:**
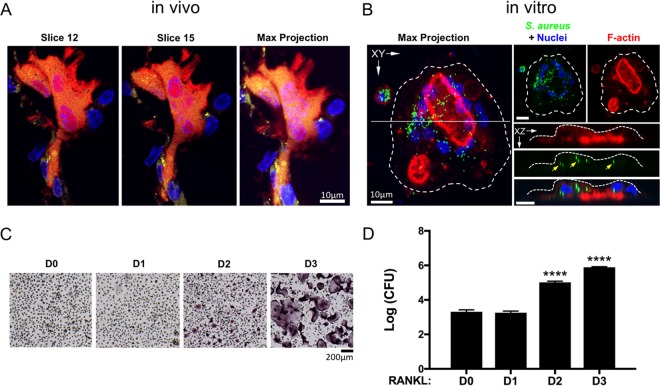
S. aureus resides within osteoclasts (OCs) *in vivo* and *in vitro*. (A) Confocal microscopy of histological sections reveals internalized GFP+ S. aureus inside TRAP^Red^ OCs within mouse calvarium (yellow puncta) 24 h postinfection (hpi). (B) OCs differentiated from bone marrow macrophages (BMMs) with 3 days of RANKL and M-CSF on devitalized bone, infected, and treated with gentamicin also harbor S. aureus (green puncta) 18 hpi. (C) TRAP staining of BMMs differentiated toward OCs for up to 3 days showing multiple TRAP+ mononuclear cells at day 2 (D2) and numerous TRAP+ multinuclear fully differentiated OCs at D3. (D) Enumerated CFU grown on tryptic soy agar from lysates of BMMs differentiated toward OCs for up to 3 days and infected with S. aureus for 18 h, with gentamicin killing of extracellular bacteria. Values that are significantly different from the values at D0 by one-way ANOVA with Tukey’s *posthoc* test are indicated as follows: ****, *P* < 0.0001. *n* = 3 technical replicates, representative of >5 biological replicates.

Although previous studies have shown that S. aureus invasion of osteoblasts leads to RANKL expression and robust OC recruitment at sites of bone infection, it remains unclear whether OCs are cellular targets for intracellular infection. To demonstrate the intracellular presence of S. aureus within OCs *in vivo*, we injected green fluorescent protein (GFP)-expressing S. aureus (USA300 lineage strain LAC) subcutaneously over the periosteum of calvaria in TRAP-tdTomato (herein referred as TRAP^Red^) reporter mice (39). Prior to microbial challenge, RANKL was injected daily over the calvaria for 5 days to recruit OCs and their precursors to the subsequent site of infection. At 24 h postinfection (hpi), calvaria were harvested, and histological sections were examined by confocal microscopy to visualize bacteria residing within OCs, since both are fluorescently labeled. As shown in Fig. 1A, GFP-expressing S. aureus can clearly be found localized within TRAP^Red^ OCs. Murine OCs were also generated on bone slices *in vitro*, then infected with GFP-labeled S. aureus, and treated with gentamicin to destroy extracellular bacteria. The cells were imaged 18 h later, and bacteria were again found intracellularly within OCs (Fig. 1B).

In order to demonstrate whether the observed intracellular bacteria were viable, we used a gentamicin-based protection assay. Murine bone marrow macrophages (BMMs) were differentiated with RANKL for up to 3 days (Fig. 1C) and infected with S. aureus for 30 min at a multiplicity of infection (MOI) of 1:1, after which extracellular bacteria were killed by the addition of gentamicin for 1 h. Infected cells were lysed after 16.5 h of additional culture (at 18 hpi), and CFU were enumerated. While exposure of BMMs to RANKL for 1 day had no effect on the number of bacteria recovered, 2 days of differentiation in RANKL (lineage-committed TRAP-positive [TRAP+] preosteoclasts [pre-OCs]) led to ∼100-fold-increased bacterial load, and 3 days of RANKL (fully differentiated OCs) caused an ∼500-fold change, compared to no RANKL (undifferentiated BMMs) (Fig. 1D). In order to preclude any confounding effects that may be specific to gentamicin treatment in our assay, we repeated the 18 hpi CFU assay during OC differentiation with lysostaphin as the bactericidal agent instead of gentamicin. We found that the patterns of increased intracellular bacterial load in OCs at 18 hpi were the same in our lysostaphin protection assay as with our gentamicin protection assay (see Fig. S1 in the supplemental material).

10.1128/mBio.02447-19.1 FIG S1 Osteoclasts show increased intracellular bacterial load 18 hours after S. aureus infection by lysostaphin protection assay. Colony-forming units (CFU) from lysates of OCs differentiated for 0, 2, or 3 days (D0, D2, or D3, respectively) and subjected to the lysostaphin protection assay after infection with S. aureus. Lysates were harvested at 18 hours postinfection. **, *P* = 0.0014; ****, *P* < 0.0001 by one-way ANOVA with Tukey’s posthoc analysis. *n* = 3 biological replicates. Download FIG S1, TIF file, 0.04 MB. Copyright © 2019 Krauss et al. 2019 Krauss et al. This content is distributed under the terms of the Creative Commons Attribution 4.0 International license.

### S. aureus proliferates within osteoclasts *in vitro*.

**FIG 2:**
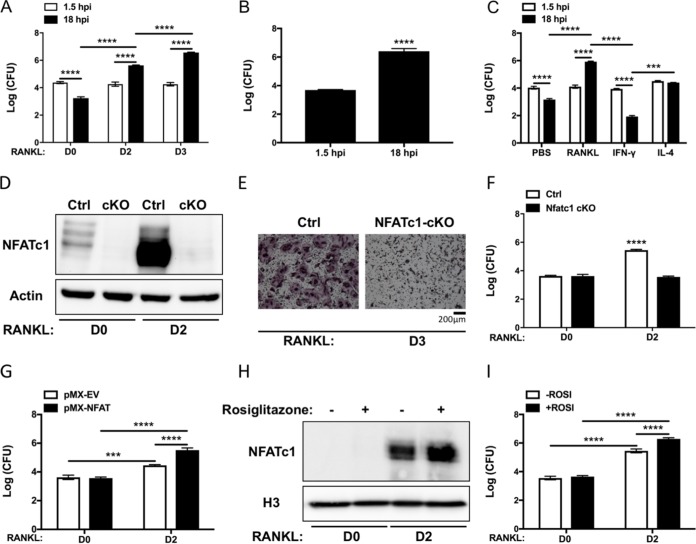
S. aureus proliferates within OCs *in vitro* in a RANKL-induced Osteoclastogenesis-dependent manner. BMMs were treated with RANKL for up to 3 days and subjected to the gentamicin protection assay. (A) CFU from lysates of infected cells after OC differentiation for 0, 2, or 3 days. Cells were lysed immediately after gentamicin exposure (1.5 hpi) or after an additional 16.5 h in osteoclastogenic media (D2, D3) or control media (D0). *n* = 3 technical replicates, representative of >5 biological replicates. (B) CFU from lysates of infected human CD14^+^ monocytes isolated from peripheral blood and differentiated into OCs for 3 days before infection. *n* = 3 biological replicates. (C) CFU from lysates of infected BMMs after exposure for 2 days to either PBS, RANKL, IFN-γ, or IL-4. *n* = 3 technical replicates, representative of 3 biological replicates. (D) BMMs harvested from NFATc1 conditional knockout animals (cKO) show no basal (D0) or induced (D2) NFATc1 protein by Western blotting compared to littermate control mice BMMs (Ctrl). (E) TRAP staining after 3 days of RANKL exposure demonstrates failure of NFATc1-cKO BMMs to form TRAP+ multinuclear OCs. (F) CFU from lysates of Ctrl or NFATc1 cKO BMMs differentiated into OCs for 0 or 2 days and subjected to the gentamicin protection assay. All bars represent 18 h postinfection (hpi). White bars, Ctrl; black bars, NFATc1 cKO cells. (G) CFU from lysates of wild-type (WT) BMMs transfected with empty vector (pMX-EV [white bars]) or NFATc1-overexpressing vector (pMX-NFAT [black bars]) at 18 hpi, showing positive effect of NFATc1 at D2. (H) Rosiglitazone treatment of WT BMMs increases NFATc1 induction more than RANKL alone as measured by Western blotting of nuclear extracts. H3, histone 3 antibody. (I) CFU from lysates of WT BMMs treated with Rosiglitazone (+ROSI [black bars]) or untreated (-ROSI [white bars]) at 18 hpi. *n* = 3 biological replicates. **, *P* < 0.01; ***, *P* < 0.001; ****, *P* < 0.0001 by two-way ANOVA with Tukey’s *posthoc* test (A, C, F, G, and I) or Student’s *t* test (B).

BMMs have innate immune activity and have previously been shown to phagocytose and kill intracellular bacteria (36). Using the gentamicin protection assay, we confirmed that our BMMs behave similarly, finding that the number of intracellular S. aureus at 18 hpi is ∼10-fold lower than at 1.5 hpi, immediately after removal of extracellular bacteria by antibiotic treatment (Fig. 2A, day 0 [D0]). In contrast, bacterial recovery from RANKL-treated cultures (day 2 [D2] or day 3 [D3]) demonstrated a dramatic increase between 1.5 and 18 hpi, suggesting that S. aureus can proliferate within OCs. There was no difference in the ability of any of the cultures to internalize S. aureus, as the 1.5 hpi bacterial counts were similar across groups. In order to rule out potential differential effects of OCs grown on culture-treated plastic, we confirmed similar results in OCs grown and infected on bone chips *in vitro* (Fig. S2). Next, to establish whether these observations were merely an artifact of murine OCs, we infected primary human OCs generated from peripheral blood CD14^+^ monocytes. Consistent with the data in the mouse system, human OCs significantly promote intracellular replication of S. aureus (Fig. 2B).

10.1128/mBio.02447-19.2 FIG S2 OCs grown on bone chips *in vitro* allow S. aureus replication similar to OCs grown on plastic. Colony-forming units (CFU) from lysates of OCs grown on bone chips for 5 days and subjected to the gentamicin protection assay after infection with S. aureus, harvested at 1.5 or 18 hours postinfection (hpi). **, *P* < 0.01 by *t* test. *n* = 3 biological replicates. Download FIG S2, TIF file, 0.04 MB. Copyright © 2019 Krauss et al. 2019 Krauss et al. This content is distributed under the terms of the Creative Commons Attribution 4.0 International license.

Since the antimicrobial properties of myeloid cells are altered by cytokine exposure, we sought to determine whether RANKL was unique in its ability to promote intracellular bacterial growth. To address this question, we polarized BMMs for 2 days with gamma interferon (IFN-γ), toward an antimicrobial M1 phenotype, or with interleukin 4 (IL-4), toward an M2 phenotype expected to have reduced antimicrobial defense, and then compared these to RANKL-stimulated BMMs (i.e., pre-OCs). In all cases, levels of bacteria were similar at 1.5 hpi, indicating consistent internalization (Fig. 2C). IFN-γ treatment led to higher levels of killing than observed in control phosphate-buffered saline (PBS)-treated BMMs, as expected for M1-polarized cells. In the IL-4-stimulated, M2-polarized cells, we found that S. aureus bacteria are present at the same levels 1.5 and 18 hpi, indicating persistence rather than intracellular killing or proliferation. Thus, RANKL initiates a distinct program that alters the antimicrobial profile of BMMs as they differentiate toward OCs.

### Proliferative capacity of S. aureus within osteoclasts is NFATc1 dependent in response to RANKL.

Having demonstrated that S. aureus proliferates robustly within differentiated OCs, we next wanted to determine whether deficiency of NFATc1, the master transcriptional regulator of OC formation, would modulate intracellular bacterial levels in response to RANKL. To this end, *Nfatc1^fl/fl^* mice were mated to an inducible *Mx1-cre* transgenic line, and poly(I·C) was used to conditionally delete *Nfatc1* 1 month prior to harvest of BMMs (Fig. 2D). Consistent with the critical function of NFATc1 in driving OC differentiation (Fig. 2E), we find that S. aureus was unable to replicate in NFATc1-deficient cells in response to RANKL stimulation (Fig. 2F, D2 condition). In contrast, we found no difference in the microbial load between unstimulated control and NFATc1-deficient BMMs, a result consistent with the low basal expression of NFATc1 in the absence of RANKL (Fig. 2D).

Because we found that RANKL-induced NFATc1 promotes intracellular replication of S. aureus, we evaluated whether forced expression of this molecule would further enhance microbial expansion. For this purpose, we cloned *Nfatc1* cDNA into the pMX retroviral vector and transduced wild-type (WT) BMMs. After blasticidin selection, transduced cells were cultured with macrophage colony-stimulating factor (M-CSF) alone or under osteoclastogenic conditions and challenged with S. aureus. Using this gain-of-function approach, we demonstrate that ectopic expression of NFATc1, which accelerates OC differentiation, significantly increased microbial burden compared to cells transduced with empty vector under osteoclastogenic conditions (Fig. 2G). Surprisingly, overexpression of NFATc1 in BMMs, in the absence of RANKL signaling, failed to support replication, as the microbial loads were comparable to those of the empty vector control. Similarly, treatment with rosiglitazone, a peroxisome proliferator-activated receptor-γ agonist that augments RANKL-induced NFATc1 levels when combined with RANKL (Fig. 2H), led to a significant increase in intracellular microbial accumulation relative to untreated OCs (Fig. 2I). However, in the absence of RANKL, NFATc1 expression was not induced, and intracellular microbial counts were comparable between rosiglitazone-treated and untreated BMMs (Fig. 2I). Thus, using both genetic and pharmacological approaches, intracellular proliferation of S. aureus within OCs is NFATc1 dependent in response to RANKL stimulation.

### Alternative NF-κB promotes intracellular expansion of S. aureus.

**FIG 3:**
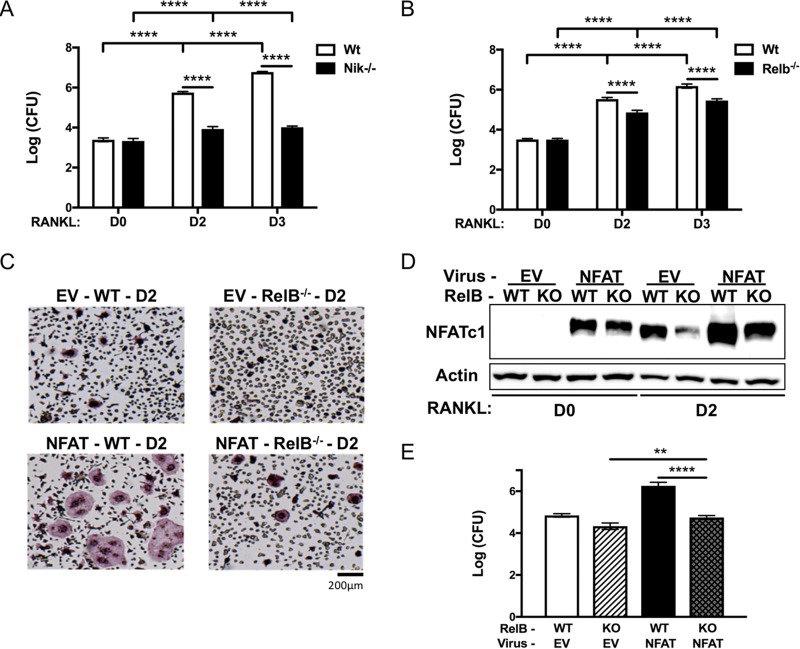
Alternative NF-κB in OCs promotes intracellular S. aureus replication *in vitro*. (A and B) BMMs harvested from NIK knockout mice (Nik^−/−^ [black bars]) (A) and RelB knockout mice (Relb^−/−^ [black bars]) (B) were differentiated into OCs for 0, 2, or 3 days and subjected to the gentamicin protection assay. CFU of intracellular S. aureus proliferation were examined at 18 hpi. (C) TRAP-stained images of WT or Relb^−/−^ BMMs transduced with empty vector (EV) or NFATc1-overexpressing virus (NFAT) and differentiated for 48 h. (D) Western blot of NFATc1 protein expression from WT or Relb^−/−^ (KO) BMMs transduced with EV or NFAT and differentiated with RANKL for 0 (D0) or 2 (D2) days. (E) WT or Relb^−/−^ (KO) cells transduced with EV or NFAT virus, differentiated in RANKL for 3 days, infected with S. aureus, and subjected to the gentamicin protection assay. Bars represent CFU of lysates at 18hpi. *n* = 3 biological replicates. Statistical significance: in panels A and B, ****, *P* < 0.0001 by two-way ANOVA with Tukey’s *posthoc* test; in panel E,**, *P* < 0.01; ****, *P* < 0.0001 by one-way ANOVA with Tukey’s *posthoc* test.

Although our data demonstrate a clear role for NFATc1 in facilitating intracellular growth of S. aureus in OCs, we found that microbial burden was unaltered in NFATc1-overexpressing BMMs compared to empty vector controls, suggesting that other RANKL-induced signaling pathways are also essential for the effect. Previously, our group has demonstrated that the alternative NF-κB signaling pathway is highly upregulated by RANKL stimulation and promotes OC formation (34, 35, 40). In order to investigate the importance of alternative NF-κB signaling in promoting S. aureus intracellular expansion, we utilized cells deficient in the alternative NF-κB central upstream kinase NIK or the downstream transcription factor RelB. Loss of either NIK or RelB significantly decreases the ability of S. aureus to replicate intracellularly (Fig. 3A and B).

NFATc1 levels are significantly reduced with RelB deficiency, and retroviral overexpression of NFATc1 can rescue deficient OC formation that results from the loss of RelB (35). Therefore, we next determined whether ectopic expression of NFATc1 could restore the ability of S. aureus to replicate in RelB-deficient cells. Restoration of NFATc1 to empty vector WT levels rescued osteoclastic differentiation (Fig. 3C and D), and this resulted in the restoration of the microbial burden to empty vector WT levels in RelB knockout cells under osteoclastogenic conditions (Fig. 3E). These data highlight the importance of alternative NF-κB and NFATc1 signaling in mediating intracellular S. aureus replication.

### S. aureus can proliferate within individual OCs to varying degrees.

**FIG 4:**
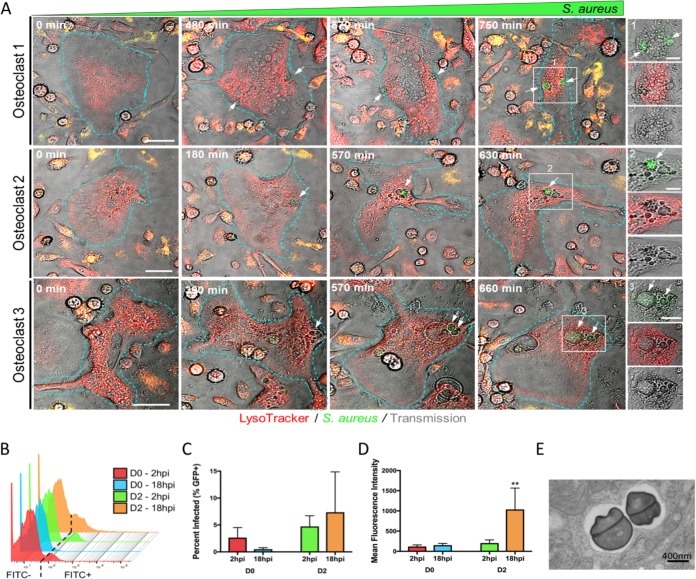
S. aureus proliferates within individual OCs to different magnitudes. (A) Images depict representative OCs infected with GFP+ S. aureus (green) and lysosomes labeled with LysoTracker (red). Outlines indicate cell boundaries. White arrows track the formation of intracellular GFP+ S. aureus within vesicles over the indicated time frames. Insets represent higher magnified images of enumerated white boxes. Bars = 20 μm and 5 μm within image and insert panels, respectively. (B) Offset histogram of flow cytometric data from infected cells differentiated in RANKL for 0 or 2 days and then infected with GFP+ S. aureus at an MOI of 1:1. Infected cells were detected by an increased signal in the FITC channel. The threshold for FITC+ is depicted by the dashed line, based on cells infected with GFP-labeled bacteria. *n* = 3 biological replicates. (C) Infected cells (GFP+) are shown as a percentage of total cells, as measured via flow cytometry. (D) Mean fluorescence intensity (MFI) as measured from the FITC+ fraction of cells via flow cytometry. **, *P* < 0.01; ****, *P* < 0.0001 by two-way ANOVA with Tukey’s *posthoc* test. (E) Transmission electron micrograph of dividing S. aureus in a membrane-bound compartment inside an OC.

The preceding experiments show that OCs can permit the growth of S. aureus in a RANKL-induced, osteoclastogenic-dependent manner at the population level (CFU assay). In order to monitor the bacteria within individual OCs over time, we utilized time-lapse fluorescence microscopy to visualize OCs infected with GFP-positive (GFP+) S. aureus. Monitoring three representative OCs over the course of 10 to 12 h, the GFP+ signal clearly increases with time (Fig. 4A, white arrows; see also Movie S1 in the supplemental material). Interestingly, the growing clusters of GFP+ bacteria do not appear to colocalize with phagolysosomes but rather seem to localize within inclusion vesicles that are devoid of LysoTracker staining (Fig. 4A, insets). Next, in order to transcend limitations in resolution and scope inherent to the time course examination of single, infected cells, we utilized flow cytometry to further explore the fate of GFP-labeled S. aureus within each cultured cell after internalization. BMMs or pre-OCs grown in RANKL for 2 days were infected at an MOI of 1:1, extracellular bacteria were killed with gentamicin as before, and then cells were fixed at 2 or 18 hpi. At this low MOI, a minority of cells bear detectable levels of GFP+ bacteria at 2 hpi, and the levels are similar for BMMs and pre-OCs (Fig. 4B and C), as we observed in the bulk CFU assay (Fig. 2A and C). By 18 hpi, the fraction of GFP+ BMMs decreased similar to previous observations, although it did not reach statistical significance. There was no statistically significant change in the percentage of GFP+ D2 pre-OCs at 18 hpi, although there was higher variability between biological replicates. Very few bacteria were detected in the culture media at 12 to 18 hpi, suggesting it is unlikely that cell lysis and new infection of adjacent cells occur at high enough frequencies to affect our readouts (see Table S1 in the supplemental material). We next plotted the mean fluorescence intensity (MFI) of the GFP+ populations in each culture (Fig. 4D). There was no change in the BMMs with time, suggesting that the few cells that were unable to clear bacteria nevertheless restrained their growth. In contrast, the MFI of GFP+ pre-OCs increased approximately fourfold between 2 and 18 hpi, indicating a greater number of bacteria per cell at the later time point. Further, the histogram (Fig. 4B) shows a population of pre-OCs at 18 hpi with a very high MFI (up 100- to 500-fold) compared to any other culture condition. Supporting our conclusion that the bacteria are expanding within RANKL-treated cells, transmission electron microscopy demonstrates dividing bacteria in mature OCs at 18 hpi (Fig. 4E). Taken together, the time-lapse imaging and flow cytometry data demonstrate that S. aureus is able to proliferate within individual OCs. Furthermore, the flow cytometry data suggest that a subset of pre-OCs permits the rapid intracellular proliferation of bacteria to a far greater degree than the majority of pre-OCs.

10.1128/mBio.02447-19.4 TABLE S1 Increased S. aureus colony formation is not the result of an increased extracellular bacterial load. Colony-forming units (CFU) from sampled media during the course of the gentamicin protection assay. Samples taken from the media of cells differentiated into OCs for 0 (D0) or 2 (D2) days at 12, 15, or 18 h postinfection (hpi). Colonies are listed as total number of colonies formed (#) and the CFU (log) after 1,000-fold dilution. Download Table S1, PDF file, 0.2 MB. Copyright © 2019 Krauss et al. 2019 Krauss et al. This content is distributed under the terms of the Creative Commons Attribution 4.0 International license.

10.1128/mBio.02447-19.5 MOVIE S1 GFP+ S. aureus signal increases over time within a single osteoclast. A time-lapse video depicts a single infected osteoclast over 810 minutes. The GFP signal of the GFP+ S. aureus appears within vacuoles and increases over that time and does not appear to colocalize with the LysoTracker Red (white arrows). Images were captured at 30-min intervals and played back at five frames per second. Download Movie S1, MOV file, 1.3 MB. Copyright © 2019 Krauss et al. 2019 Krauss et al. This content is distributed under the terms of the Creative Commons Attribution 4.0 International license.

### S. aureus within osteoclasts is not located exclusively in phagolysosomes.

**FIG 5:**
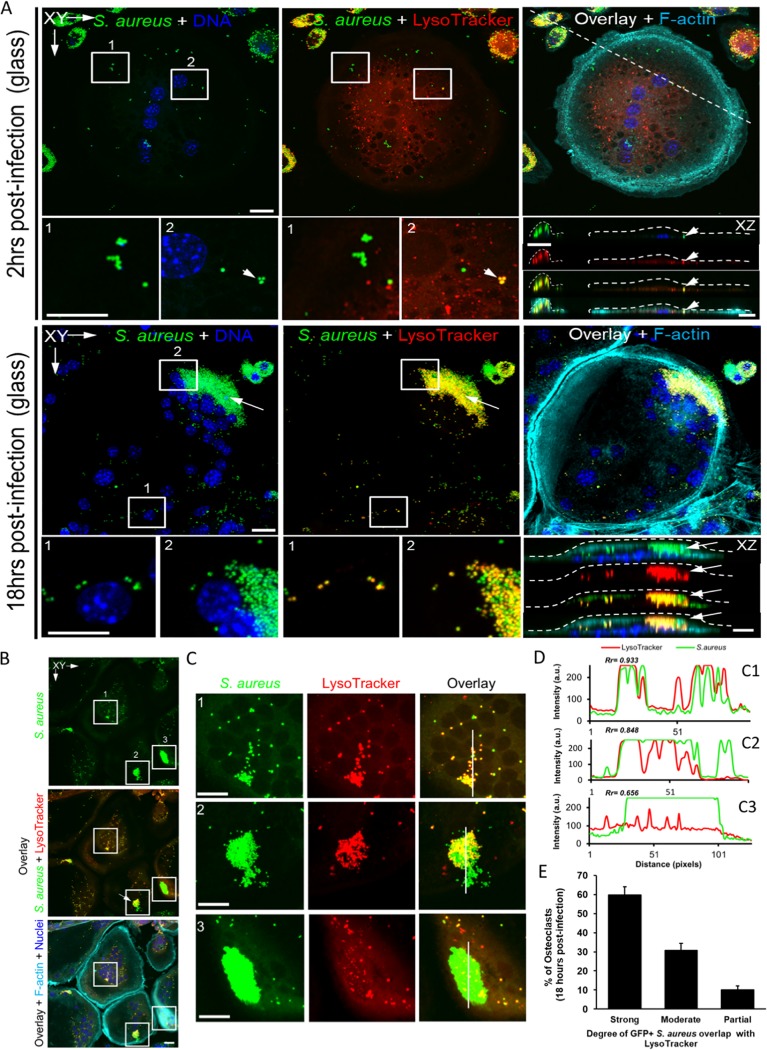
Significant percentage of OCs do not have high S. aureus colocalization with lysosomes. Confocal microscopy images of OCs at 2 or 18 h after being infected with GFP+ S. aureus. (A and B) Images show GFP+ S. aureus (green), lysosomes (LysoTracker, red), F-actin (turquoise), and nuclei (blue) within OCs. Enumerated inserts represent higher magnified images (C). (D) The cross-correlation analysis of the peaks of green (S. aureus) and red (LysoTracker) fluorescence intensity from the line scans (C1 to C3, white lines) is represented with the corresponding Pearson’s coefficient (Rr). (E) A bar graph depicting the percentage of OCs exhibiting either a strong, moderate, or partial degree of GFP+ S. aureus*-*LysoTracker colocalization as based on their respective Pearson’s coefficient (Rr). *n* = 3 biological replicates with 20 to 30 OCs counted per field. Bars = 10 μm.

Because the optical resolution is limited for live cell images, we further evaluated the apparent phagolysosome avoidance by S. aureus in OCs with endpoint confocal microscopy at 18 hpi, using GFP+ bacteria and LysoTracker red staining. Three-dimensional reconstructions show a modest amount of GFP+ S. aureus inside the cell imaged at 2 hpi (Fig. 5A, top), whereas the amount is higher in the cell imaged at 18 hpi (Fig. 5A, bottom). We see similar results when OCs are grown and infected on bone slices (Fig. 1 and Fig. S3). However, analogous to the wide range in fluorescence intensity of infected D2 pre-OCs demonstrated by flow cytometry (Fig. 4B), the number of bacteria in each mature OC was variable across the entire population in a particular culture. There are numerous examples of bacteria not colocalizing with phagolysosomes (Fig. 5A to C, white arrows and insets). Interestingly, in cells with a low bacterial load (Fig. 5C, box 1), there appears to be a high degree of colocalization between the bacteria and phagolysosomes (Fig. 5C and D). Comparatively, cells harboring very large clusters of bacteria (Fig. 5B and C, boxes 2 and 3) seem to have more bacteria that do not appear to reside in acidified phagolysosomal compartments (Fig. 5C), although the degree varies. In order to quantify the colocalization of the fluorescent signal peaks from the green GFP+ bacteria with the red LysoTracker-stained phagolysosomes, we evaluated the Pearson’s colocalization correlation coefficients (Rr) generated from line scans taken within each cell (Fig. 5C and D). Using the Pearson’s coefficients (Rr) generated from multiple OCs (20 to 30 per field, per experiment, over three experiments), we find that OCs separate into three categories of colocalization between S. aureus and phagolysosome staining. About 60% of OCs exhibit a strong degree of colocalization, while the other 40% exhibit only a moderate (30%) or partial (10%) colocalization (Fig. 5E). These data suggest phagolysosomal evasion or destruction as potential mechanisms underpinning S. aureus intracellular proliferation within OCs. Furthermore, as both flow cytometry and confocal microscopy show a small percentage of OCs with very high bacterial loads, it is possible that a subpopulation of OCs is responsible for the majority of S. aureus proliferation observed at the population level.

10.1128/mBio.02447-19.3 FIG S3 S. aureus in OCs on bone is not exclusively in lysosomes. Confocal microscopy images of BMMs differentiated into OCs on bone slices and then infected with GFP+ S. aureus, imaged at 2 hpi or 18 hpi in the indicated planes. GFP+ S. aureus (green), lysosomes (LysoTracker [red]), F-actin (turquoise), and nuclei (blue). OC at 18 hpi is the same as shown in Fig. 1B, with LysoTracker added and different pseudocolor. Scale bars = 10μm. Download FIG S3, TIF file, 2.7 MB. Copyright © 2019 Krauss et al. 2019 Krauss et al. This content is distributed under the terms of the Creative Commons Attribution 4.0 International license.

## DISCUSSION

OM is a common and debilitating infection, with associated osteolysis causing pain and pathological fractures. To this point, most of the work examining OCs in the context of OM has focused on the stimulatory effect of S. aureus on OC activity. In this study, we utilized a low multiplicity of infection (MOI) of 1 to 10 which may reveal host-pathogen interactions that are masked by much higher bacterial loads, as different S. aureus inocula have been shown to have differential effects on the immune response and infection progression (41). With this approach, we have shown that OCs are a target of S. aureus infection, *in vivo* and *in vitro*, providing the bacteria a replicative niche. Unlike their progenitors, OCs are unable to completely confine internalized S. aureus to phagolysosomes, the likely cause of their failure to eliminate the bacteria. Thus, the direct interactions between OCs and S. aureus may play an important role in the progression of OM beyond bone loss, affecting the survival and proliferation of the pathogens.

The ability of S. aureus to proliferate after internalization was dependent on prior RANKL stimulation of the host cell for 2 days, the point at which cells become positive for TRAP and are considered to be committed to the OC lineage. Complete differentiation into multinucleated OCs, whether on plastic or bone, was associated with an even higher level of intracellular bacteria. The effect of RANKL was unique, as polarization of the BMMs toward M2 macrophages with IL-4, which causes reduced bacterial killing, did not allow intracellular replication. Since osteoclastogenesis depends on NFATc1, it is not surprising that this transcription factor was also required for the effect of RANKL on S. aureus. Interestingly, overexpression of NFATc1 alone, which is not sufficient to cause OC differentiation, did not promote bacterial proliferation without concomitant RANKL exposure. This suggests that additional signals from RANKL/RANK are required. The alternative NF-κB pathway is activated by RANKL and is upstream of NFATc1 and other factors important for OC function (35). Deficiency of NIK, the apex kinase, results in a more severe impairment in OC differentiation relative to genetic disruption of RelB, the key transcriptional subunit (35), and likewise NIK ablation blunted S. aureus proliferation more than RelB deficiency. Furthermore, restoration of NFATc1 expression in the RelB-deficient background to WT levels also normalized both OC differentiation and bacterial loads, suggesting that in this context, NFATc1 activation is the primary mediator of bacterial handling. These data are especially interesting in light of findings from others that elucidate the importance of RANKL signaling in driving osteoclast-mediated inflammatory bone resorption (42). Overall, these findings bolster our conclusion that RANKL-induced osteoclastogenesis is necessary for the increased bacterial replication, and they add to the rigor of our studies as we have shown consistent results across different mouse colonies, genetic knockouts, and treatments.

By using a low MOI, we observed cellular heterogeneity within our cultures in both the D2 pre-OCs as well as in mature OCs. By using flow cytometry, we were able to identify the small percentage of cells that actually became infected with S. aureus and then monitor the cultures over time. Since the fraction of infected cells did not change between 1.5 and 18 hpi, the increase in fluorescence represents bacteria replicating within previously infected cells, and not replication in the media and infection of new cells. We also found that among this infected population there was a subset of cells that allowed the replication of bacteria to a much greater extent than other cells, as reflected by the “tail” of cells with a high MFI only at 18 hpi in D2 pre-OCs. In fact, this minority population of cells could be responsible for the more than 2-log_10_-unit increase in bacteria represented in the MFI by flow cytometry and CFU in the antibiotic protection assays. It is possible that differences in bacterial expansion within the pre-OCs at D2 are related to the degree of differentiation, since these primary cell cultures are somewhat heterogeneous. However, a similar variability in bacterial load was seen by confocal microscopy in mature OCs.

As OCs are differentiated from BMMs, it is important to compare the consequences of bacterial internalization between the two cell types in order to understand how OCs fail where BMMs succeed. Although S. aureus has been shown to be able to replicate within human macrophages (37, 38), typically murine BMMs effectively destroy internalized bacteria via phagolysosome acidification. For S. aureus, this is a process involving NLRP3 inflammasome activation and caspase-1 cleavage that leads to NADPH oxidase 2 (NOX2) production of reactive oxygen species (36). We discovered that mature multinucleated OCs exhibited different degrees of colocalization of S. aureus with phagolysosomes, and interestingly, the OCs with the highest intracellular bacterial loads had the lowest degree of S. aureus colocalization with phagolysosomes. By time-lapse fluorescence microscopy, we detected S. aureus in clusters only, and these were within vacuoles that excluded LysoTracker staining, likely representing the cells with the highest bacterial loads. It is possible that the ∼10% of OCs we found with only a partial degree of GFP+ S. aureus-phagolysosome colocalization also coincide with the subset of pre-OCs with very high GFP+ bacterial load via flow cytometry. Our initial ultrastructural investigation using transmission electron microscopy showed that dividing S. aureus can be found in membrane-bound compartments, but the bacterial load in the visualized cells was relatively low compared to most seen on confocal microscopy. Therefore, it is possible that bacteria replicate in the cytoplasm as well. Although under our experimental conditions, lysis of OCs is uncommon before 18 hpi, it becomes frequent by 24 hpi. Future work will focus on tracing the intracellular fate of the bacteria within OCs over time, including defining the endosomal compartments involved and whether they escape into the cytoplasm prior to cell death. Additional single-cell approaches will be required to determine which host cell pathways determine the fate of intracellular S. aureus and whether these are responsible for the observed differences between cells.

Ultimately, this work elucidates a new role for OCs in propagating infectious OM, at least in the context of S. aureus. It has previously been shown that osteoblasts release proosteoclastogenic cytokines, including RANKL, that recruit and activate OCs at the site of infection. While others have shown that S. aureus and its cellular components can promote osteoclastogenesis, OC activity, and proinflammatory cytokine release (29, 32, 43), this investigation focused on determining the infectibility of OCs. We found that it is possible for OCs to become infected and provide a replicative niche for S. aureus to proliferate and evade immune destruction and that this whole process is dependent on RANKL signaling. Although the influence of S. aureus on OC activity and cytokine production is undoubtedly important for the progression of OM lesions, we have extended the potential role for these cells to include bacterial expansion. If S. aureus promotes OC recruitment and formation and OCs can harbor bacteria from destruction, then these conditions can form a positive-feedback loop. Thus, therapies aimed at modulating the ability of OCs to shelter bacteria might provide increased efficacy in curing these difficult-to-treat infections.

## MATERIALS AND METHODS

### Reagents.

Trypticase soy broth was procured from Fisher Scientific (Hampton, NH, USA). Fetal bovine serum (FBS) and gentamicin (catalog no. 15750060) were purchased from Gibco-BRL (Grand Island, NY, USA). α-Minimal essential medium (α-MEM), anti-α-actin (A2228) antibody, rosiglitazone, polyinosinic-poly(C) [poly(I·C)], and lysostaphin (L7386) were all purchased from Sigma-Aldrich (St. Louis, MO, USA). Macrophage colony-stimulating factor (M-CSF), in the form of CMG 14-12 supernatant, and glutathione *S*-transferase RANKL (GST-RANKL) were prepared as previously described (33). Human M-CSF and Ficoll histopaque were purchased from Invitrogen (Carlsbad, CA, USA). Human CD14 magnetic beads were obtained from Miltenyi Biotec (Auburn, CA, USA). Anti-NFATc1 (7AG) antibody was obtained from Santa Cruz Biotechnology (Dallas, TX, USA), and anti-histone H3 (96C10) was obtained from Cell Signaling Technology (Beverly, MA, USA). Murine recombinant IL-4 and IFN-γ were purchased from Peprotech (Rocky Hill, NJ, USA).

### Mice.

C57BL/6 male and female mice (8 to 10 weeks old) were purchased from The Jackson Laboratory (Bar Harbor, ME, USA). *Relb^−/−^* and *Nik^−/−^* mice and their littermate controls were generated by heterozygotic mating of *Relb^+/−^* and *Nik^+/−^* mice, respectively, in a specific-pathogen-free facility as previously described (34, 40). TRAP promoter-tdTomato (TRAP^Red^) transgenic mice were generated by M. Ishii (Immunology Frontier Research Center, Osaka University, Osaka, Japan [39]), provided by J. Lorenzo, and maintained by heterozygotic mating in our specific-pathogen-free facility. Conditional knockout *Nfatc1* (*Nfatc1 cKO*) mice were provided by Julia Charles (Harvard Medical School, Boston, MA) and generated by crossing *Nfatc1 fl/fl* mice with a transgenic line expressing the Cre recombinase from a type I interferon-inducible promoter (*Mx1*-*Cre*) as previously described (44). Activation of *Mx1-Cre* was achieved by intraperitoneal injection of 0.25 ml of 1 mg/ml poly(I·C) in phosphate-buffered saline (PBS) every other day. Three rounds of injections were given, followed by a 3-week waiting period to ensure optimal *Nfatc1* deletion. Littermates lacking the *Mx1-Cre* transgene were treated identically with poly(I·C) and served as NFATc1-sufficient (*Nfatc1fl/fl*) controls. For all experiments employing genetically modified mice, results were compared to cells from sex/age-matched littermate controls.

### Osteoclast culture.

To generate osteoclasts (OCs) from enriched bone marrow macrophages (BMMs), bone marrow was harvested from the long bones of 10- to 12-week-old mice, and cells were cultured in α-MEM plus 10% FBS and a 1:10 dilution of CMG 14-12 cell supernatant (containing the equivalent of 100 ng/ml of M-CSF) for 4 days to expand BMMs. Nonadherent cells were removed by several washes in PBS, and adherent BMMs were detached with trypsin-EDTA, seeded into tissue-cultured treated plates and cultured in α-MEM plus 10% FBS containing a 1:50 dilution of CMG 14-12 cell supernatant (containing the equivalent of 20 ng/ml of M-CSF) and GST-RANKL (60 ng/ml) with medium changes every day for the indicated time periods.

For human osteoclastogenesis, peripheral blood mononuclear cells were obtained by density gradient centrifugation with Ficoll histopaque. Monocytes were isolated using anti-CD14 magnetic beads according to the manufacturer’s instructions. Human CD14^+^ cells were seeded into six-well plates at a seeding density of 1 × 10^6^ cells per well. Monocyte-derived osteoclasts were generated by culturing CD14^+^ cells for 6 days in α-MEM plus 10% FBS containing 20 ng/ml of human M-CSF and GST-RANKL. The medium was changed and cytokines were replenished every other day.

### Bacterial strains and growth conditions.

All experiments were conducted with derivatives of Staphylococcus aureus USA300 clinical isolate LAC (45). Bacterial strains were grown in Trypticase soy broth (TSB) overnight at 37°C with shaking at 225 rpm, subcultured at a dilution of 1:100, grown to mid-exponential phase (optical density at 600 nm [OD_600_] of 1.0), and centrifuged at 3,000 rpm for 10 min. The pellets were washed and resuspended with PBS to the desired concentration. To create a stable GFP+ strain of S. aureus, the region containing the *sarA* promoter driving superfolder GFP (sfGFP) was first amplified out of pCM11 (46) using primers 5′-GTTGTTTCTAGACTGATATTTTTGACTAAACCAAATG-3′ and 5′-GTTGTTGAGCTCTTAGTGGTGGTGGTG-3′ (restriction sites underlined). The resulting PCR amplification product was then ligated into the XbaI and SacI sites of pJC1111 to create pNP1. pNP1 was then chromosomally integrated into the SaPI1 site of S. aureus as previously described (47). The region encompassing the chromosomal SaPI1 integration was then transduced into S. aureus strain LAC (AH1263) (45) using phi80a. Integration of P*sarA*_sfGFP at the SaPI1 site was confirmed using primers JCO717 and 719 (47).

### Antibiotic protection assays.

Infection of BMMs and OCs by S. aureus was quantified by determining the number of CFU recovered from antibiotic treatment using a gentamicin protection assay as previously described (37, 38, 48). Briefly, cells were seeded at 5 × 10^5^/well in six-well plates and cultured in α-MEM plus 10% FBS containing a 1:50 dilution of CMG 14-12 cell supernatant (containing the equivalent of 20 ng/ml of M-CSF) in the presence or absence of GST-RANKL for the designated time of osteoclastic differentiation as described above. To determine the level of intracellular survival, the cells were infected for 30 min at an MOI of 1:1 (final dose of 5 × 10^5^/well) at 37°C in 5% CO_2_, washed twice in PBS, and cultured in media (α-MEM plus M-CSF with or without RANKL) containing antibiotic (gentamicin at a final concentration of 0.3 mg/ml or lysostaphin at a final concentration of 20 μg/ml) for 1 h to kill extracellular bacteria. Cells were washed twice in PBS to remove antibiotic and lysed in sterile, ice-cold ultrapure H_2_O for the 1.5 h time point (1.5 hpi). For the 18 h time point (18 hpi), culture media (α-MEM plus M-CSF with or without RANKL) were replaced after PBS washes, and infection was continued to 18 h postinfection prior to hypotonic lysis as described above. Lysates were 10-fold serially diluted, plated on TSB solidified with 1.5% agar (TSA), and incubated overnight at 37°C, and CFU were enumerated. Controls for antibiotic killing of S. aureus were included in all experiments by plating supernatant on TSA and inspecting for colonies after overnight incubation at 37°C.

### Flow cytometric assays.

For flow cytometry, cells were seeded at 5 × 10^5^/well in six-well tissue culture treated plates, as described above. Cells were challenged with GFP-expressing S. aureus at an MOI of 1:1 for 30 min, and the extracellular bacteria was killed by the addition of gentamicin to the media for 1 h. Cells were washed twice in PBS to remove antibiotic, and then the medium was replenished. The cells were then harvested at 2 h (2 hpi) or 18 h (18 hpi). Cells were detached with trypsin-EDTA and washed twice in PBS to remove nonadherent bacteria, and extracellular fluorescence (reflecting attached but uninternalized bacteria) was quenched with 0.2% trypan blue as previously described (48) Cells were washed twice in PBS, fixed in 90% methanol for 30 min at 4°C, and analyzed via flow cytometry (percent fluorescein isothiocyanate [FITC]-positive cells and mean fluorescence intensity of FITC-positive population), using a BD LSR-II flow cytometer in PBS containing 2 mM EDTA and 2% FBS. The data were analyzed using Flow Jo v 10.5.3.

### Confocal microscopy.

Laser scanning confocal microscopy of mouse calvaria was performed to assess microbial uptake by osteoclasts *in vivo*. Prior to infection, RANKL (2 mg/kg body weight) was injected over the periosteum of calvaria of TRAP^Red^ reporter mice once a day for 5 days. Subsequently, 10^7^ GFP-expressing S. aureus bacteria were suspended in 100 μl PBS and subcutaneously injected over the periosteum of the calvaria. At 24 h postinfection, calvariae were harvested, fixed overnight in 4% paraformaldehyde at room temperature, and washed six times with PBS in 15-min intervals. Calvariae were next decalcified in 14% free acid EDTA for 3 days under continuous agitation, infiltrated in 30% sucrose overnight at 4°C, followed by embedding in OCT medium and cut into 10-μm-thick sections in a coronal orientation. Tissue sections were then mounted in Prolong Gold Antifade with 4′,6′-diamidino-2-phenylindole (DAPI) and cured for 48 h. For imaging of *in vivo* bacterial uptake, optical sectioning was performed by using Nikon A1RSi confocal microscope (Nikon Instruments Inc., NY, USA). Images were collected using an oil immersion 100× objective lens (CFI Plan Apo Lambda 100× Oil; Nikon Instruments Inc.) with 0.5-μm z-steps. For each mouse, 15 to 20 optical sections were captured in 8 to 10 different regions of interest from five separate tissue sections. Lasers with wavelengths of 405, 488, and 561 nm were used to excite fluorescence from DAPI, GFP, and tdTomato reporter probes. Line averaging and sequential scanning were employed to increase the signal-to-noise ratio and minimize spectral bleed-through, resulting in a frame acquisition time of 16 s/frame. Background subtraction was carefully utilized to reduce the contribution of tissue autofluorescence. Images were processed in Nikon NIS-Elements (Nikon Instruments Inc.) and Fiji (49).

Murine bone marrow macrophage (BMM)-derived OCs differentiated on glass coverslips or devitalized bovine bone discs were incubated with GFP-expressing S. aureus (MOI of 10) followed by gentamicin as described above and cultured for a total of 18 h. To monitor for bacterial incorporation into phagolysosomes, OCs were further incubated with the acidotrophic probe Lysotracker Red DND-99 (100 nm; Invitrogen) for 30 min prior to fixation. Cells were fixed in 4% paraformaldehyde (PFA) in PBS for 15 min at room temperature (RT), permeabilized with 0.1% Triton X-100, and stained with Alexa Fluor 647-conjugated phalloidin (1:500) and Hoechst 33258 dye (1:10,000) (Invitrogen) to visualize F-actin and nuclei, respectively. Samples were then mounted in ProLong Gold Antifade (Thermo Fisher Scientific Co.) and imaged using a Nikon A1RSi confocal microscope, equipped with a 10× (dry) lens and 60× (oil immersion) lens (Nikon Instruments Inc., NY, USA). Images were collected using the Nikon NIS-C Elements software. Cross-correlation analysis (Pearson’s, Rr) of colocalization between bacteria and phagolysosomes was performed using online ImageJ macros (NIH). To quantify the percentage of OCs with different GFP+ S. aureus-lysosome colocalization, infected OCs were enumerated from three individual experiments with 20 to 30 OCs counted per field. The three groups (strong, moderate, and partial) refer to the amount of colocalization as measured by their Pearson’s Rr coefficient values as follows: strong, Rr ≥ 0.85; moderate, Rr = 0.7 to 0.85; and partial, Rr = 0.55 to 0.7.

Time-lapse confocal microscopy images were obtained of live BMM-differentiated OCs infected with GFP+ S. aureus for 30 min and following killing of extracellular bacteria by the addition of gentamicin to the media for 1 h. Cells were washed twice in PBS to remove antibiotic, and medium was replenished. Cells were subsequently incubated with LysoTracker red for 30 min, and the medium was refreshed and then imaged under controlled atmospheric conditions (37°C and 5% CO_2_) in a Tokai Hit (Fujinomiya, Japan) Stage Top Incubator (INUG2E-TIZ) for up to 18 h by time-lapse confocal microscopy (Nikon A1RSi) using a 20× (dry) objective lens.

### Immunoblotting.

Cells were washed twice on ice with cold PBS and lysed in radioimmunoprecipitation assay (RIPA) buffer (20 mM Tris [pH 7.5], 150 mM NaCl, 1 mM EDTA, 1 mM EGTA, 1% Triton X-100, 2.5 mM sodium pyrophosphate, 1 mM β-glycerophosphate, 1 mM Na_3_VO_4_, 1 mM NaF) containing Halt protease cocktail inhibitor (Thermo Scientific, Rockford, IL, USA). After 10-min incubation on ice, lysates were centrifuged at 16,000 × *g* to pellet cellular debris, and protein concentration was determined by bicinchoninic acid (BCA) quantification assay (Bio-Rad, Hercules, CA, USA). For each sample, 30 μg of total lysate was resolved by sodium dodecyl sulfate-polyacrylamide gel electrophoresis (SDS-PAGE) and semidry transferred to a polyvinylidene difluoride (PVDF) membrane. Membranes were blocked for 1 h in 5% milk in Tris-buffered saline (TBS) containing 0.1% Tween and probed with respective primary antibodies overnight at 4°C with continuous agitation, followed by 1-h incubation at room temperature with secondary HRP-conjugated antibodies. Proteins were detected using WesternBright Quantum HRP substrate (Advansta, Menlo Park, CA, USA) and visualized with Genesnap using the Syngene Imaging System (Synoptics, Frederick, MD, USA).

### Retroviral transduction.

NFATc1-pMX construct was transiently transfected into Plat-E packaging cells by calcium phosphate precipitation method as previously described (35, 50). Viral supernatant was harvested 48 h posttransfection and used to infect BMMs for 48 h in the presence of M-CSF equivalent (20 ng/ml) and 4 μg/ml Polybrene, followed by selection in blasticidin for 3 days before culture with 20 ng/ml M-CSF equivalent and GST-RANKL (30 ng/ml).

### Statistical analysis.

All data were represented as means with standard deviation. Comparisons between groups of the CFU from human OCs (Fig. 2B) were analyzed by Student’s *t* test. Comparisons between groups of CFU from 18 hpi OCs from different days of RANKL treatment (Fig. 1D) or NFATc1 overexpression (Fig. 4E) were analyzed by one-way analysis of variance (ANOVA) with Tukey’s multiple-comparison *posthoc* test (GraphPad InStat). All other data analyzed by two-way ANOVA with Tukey’s multiple-comparison *posthoc* test, and *P* < 0.05 was taken as significant.

### Ethics statement.

All animal procedures were approved by Washington University Institutional Animal Care and Use Committees (IACUC protocol 20170025), in compliance with the established federal and state policies outlined in the Animal Welfare Act (AWA) and enforced by the U.S. Department of Agriculture (USDA), Animal and Plant Health Inspection Service (APHIS), USDA Animal Care.
