# Supplementary material for: Staphylococcus aureus Infects Osteoclasts and Replicates Intracellularly
Source: mBio. 2019 Oct 15;10(5):e02447-19. doi: 10.1128/mBio.02447-19 (PMC6794488; doi:10.1128/mBio.02447-19)
Supplement: TABLE S1 [file mBio.02447-19-st001.pdf]

CFU assay on sampled media at designated hours post-infection (hpi)			
D0	hpi	Colony #	CFU (log)
	12	0	0
	15	0	0
	18	0	0
D2	hpi	Colony #	CFU (log)
	12	0	0
	15	1	1
	18	0	0
