# Supplementary figures and images for: Staphylococcus aureus Infects Osteoclasts and Replicates Intracellularly
Source: mBio. 2019 Oct 15;10(5):e02447-19. doi: 10.1128/mBio.02447-19 (PMC6794488; doi:10.1128/mBio.02447-19)

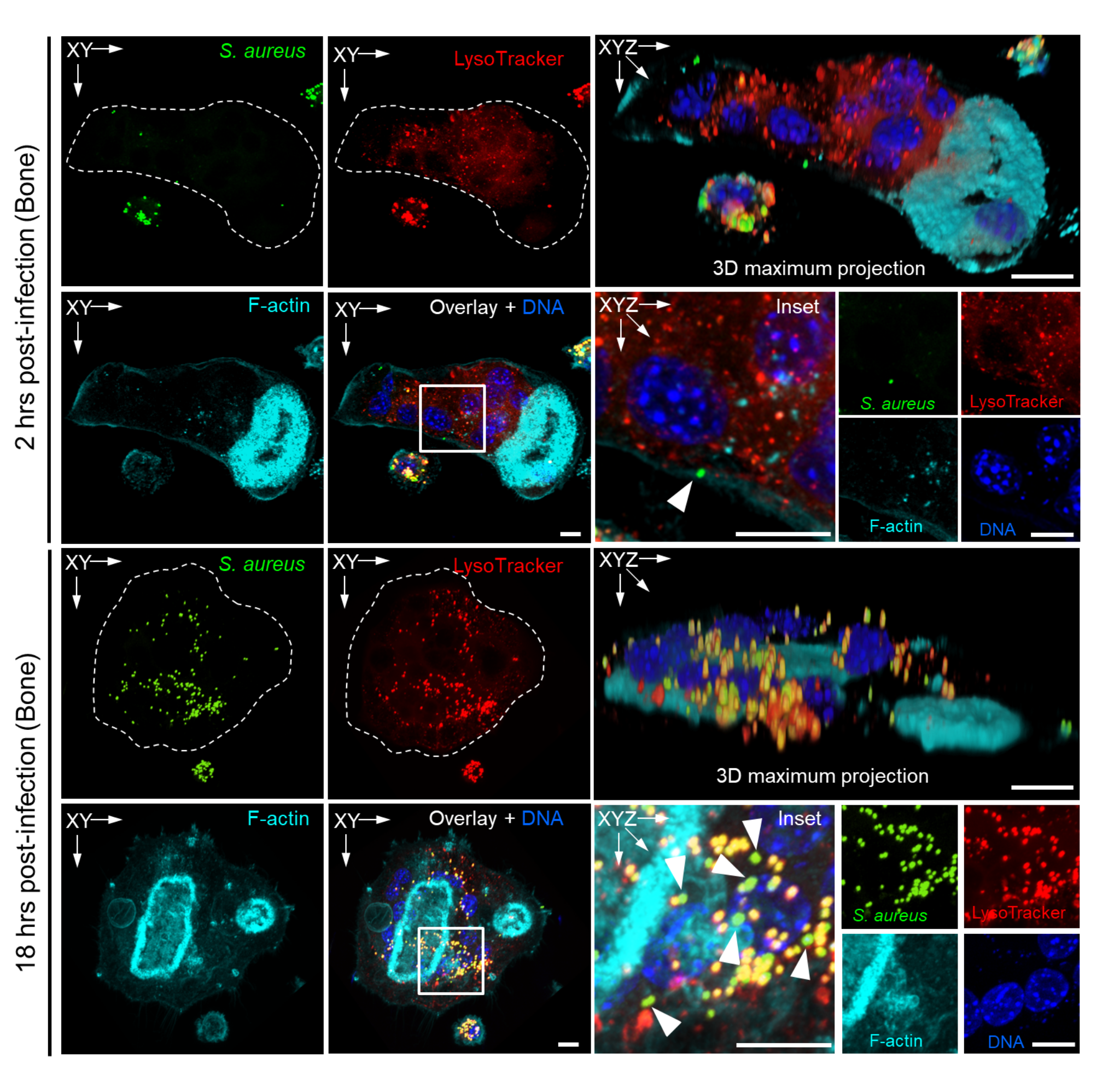

Supplement: FIG S3 [file mBio.02447-19-sf003.tif]
